# Heat Transfer Modeling of Oriented Sorghum Fibers Reinforced High-Density Polyethylene Film Composites during Hot-Pressing

**DOI:** 10.3390/polym13213631

**Published:** 2021-10-21

**Authors:** Chusheng Qi, Jinyue Wang, Vikram Yadama

**Affiliations:** 1 MOE Key Laboratory of Wood Material Science and Utilization, Beijing Forestry University, Beijing 100083, China; wjy123@bjfu.edu.cn; 2 Department of Civil & Environmental Engineering and Composite Materials and Engineering Center, Washington State University, Pullman, WA 99164, USA; vyadama@wsu.edu

**Keywords:** wood–plastic composites, heat transfer modeling, sweet sorghum, high-density polyethylene, hot-pressing

## Abstract

A one-dimensional heat transfer model was developed to simulate the heat transfer of oriented natural fiber reinforced thermoplastic composites during hot-pressing and provide guidance for determining appropriate hot-pressing parameters. The apparent heat capacity of thermoplastics due to the heat of fusion was included in the model, and the model was experimentally verified by monitoring the internal temperature during the hot-pressing process of oriented sorghum fiber reinforced high-density polyethylene (HDPE) film composites (OFPCs). The results showed that the apparent heat capacity of HDPE accurately described its heat fusion of melting and simplified the governing energy equations. The data predicted by the model were consistent with the experimental data. The thermal conduction efficiency increased with the mat density and HDPE content during hot-pressing, and a higher mat density resulted in a higher mat core temperature. The addition of HDPE delayed heat transfer, and the mat had a lower core temperature at a higher HDPE content after reaching the melting temperature of HDPE. Both the experimental and simulated data suggested that a higher temperature and/or a longer duration during the hot-pressing process should be used to fabricate OFPC as the HDPE content increases.

## 1. Introduction

Natural fibers reinforced thermoplastic composites have been commercially developed to take advantage of the low cost and high strength and stiffness of natural fibers to reinforce the matrix. In addition, these composites save petrochemical polymers and are almost climate-neutral as natural fibers absorb exactly the amount of harmful greenhouse gases from the atmosphere during their growth [1]. Hot-pressing or compression molding between two heated platens is commonly used to fabricate natural fiber composite panels because it can be used to produce final products with higher fiber loading and large size fibers/fiber bundles compared with extrusion and injection moulding. In our previously carried out studies [2,3,4], oriented sorghum fiber reinforced thermoplastic film composites (OFPCs) were manufactured with a modulus of rupture of 37.2 MPa, a modulus of elasticity of 4.7 GPa, and a thickness swelling of 2.8%. The OFPC can be used as indoor and outdoor building materials with excellent water resistance properties and without formaldehyde release. During the hot-pressing, a loosely-formed mat was compressed to its final thickness under high temperature and pressure. Heat is transported by conduction from the hot platens to the mat surface and then to the interior of the mat by conduction and convection. The hot press temperature and duration are two critical parameters for fabricating OFPCs, and modeling their heat transfer will help to understand the influence of changing these two parameters on the fabrication process and product quality.

Heat and mass transfer during hot-pressing of natural fiber composites with thermosetting resins have been investigated by many researchers [5,6,7]. However, thermoplastics have different heat characteristics than thermosetting resins. Thermoplastics will absorb heat and begin to soften below their melting temperature and flow under pressure above their melting point. Their heat capacity and conductivity change with temperature and show different trends before and after the melting point. In comparison, thermosetting resins or thermosets release heat and solidify during curing. Therefore, natural fiber and thermoplastic composites display different heat transfer characteristics than natural fiber composites bonded with thermosets. This primary difference prevents heat and mass transfer models for natural fiber composites with thermosets directly applied to natural fiber and thermoplastic composites.

Thermoplastic materials undergo a phase change, and the heat transfer is quite challenging to handle owing to the presence of solid–liquid surfaces [8]. Solving the energy equations for the liquid and solid phases separately [9] and solving it simultaneously using the enthalpy methods [10,11] are typically two approaches to tackle this challenge. Mantell and Springer [12] developed a model including three submodels, namely, the thermo-chemical model, consolidation and bonding model, and stress and strain model, to simulate the processing of thermoplastic matrix composites. Xiong et al. [13] recently developed a model to describe the consolidation behavior of thermoplastic composite prepregs during the thermoforming process based on a generalized Maxwell approach. These studies treated the melting and crystallization of thermoplastics as a complicated process, and heat absorption or generation rates were involved in their energy equation. Woo et al. [14] reported that the effective heat capacity of thermoplastics could be used to replace their heat absorption or generation during melting and crystallization. The use of effective heat capacity can simplify the energy equation to obtain its numerical solution. Still, such a method should be verified in the manufacture of natural fiber reinforced thermoplastic composites. Thermal conductivity is another critical parameter to simulate the heat transfer of natural fiber reinforced thermoplastic composites. This study will directly apply the results of our previous study about the thermal conductivity of OFPCs [3].

Many previous studies have shown that the fiber moisture content can significantly affect the heat and mass transfer of natural fiber-based composites that use thermosetting resins during hot-pressing [7,15], because water is vaporized and transfers heat from high-temperature to low-temperature regions through convection under pressure. For the fabrication of OFPC in this study, HDPE film layers inside the mat served as a barrier for vapor diffusion from the surface into the core [2]. Additionally, HDPE is hydrophobic and is incompatible with the hydrophilic sorghum fiber, and more moisture would interfere with mechanical interaction between these two materials. Therefore, oven-dried sorghum fiber was used, and the effect of moisture content on heat transfer of OFPC was ignored in this study.

As a thermoplastic and phase-change material, high-density polyethylene was assumed to gradually melt and flow into the gaps between sorghum fibers during hot-pressing. The convection caused by HDPE flow is limited, and ignored because most of the HDPE stayed in place according to previous research [4]. Our previous study showed that the vertical density profile of OFPC was not U-shaped or M-shaped, but displayed rather a zigzag fluctuation [2]. To simplify the model, a homogenous mat was assumed here as the HDPE layer was very thin.

This research aimed to develop a mathematical model capable of simulating the heat transfer of natural fiber reinforced thermoplastic composites using the apparent heat capacity of thermoplastics. The data obtained from the model were used to optimize the hot-press parameters of OFPC.

## 2. Materials and Methods

### 2.1. Materials

Extracted sweet sorghum bagasse (referred to as sorghum fiber) with a length of 20–100 mm was provided by ChloroFill, LLC in San Diego, CA, USA. Sorghum fiber was oven-dried at 103 °C to obtain a moisture content of 3% or conditioned at varied temperatures and humidity for at least one week to obtain a 6–12% moisture content. HDPE film without any additives and with a thickness of 0.1 mm and a melt flow index of 20 g/10 min at 190 °C/2.16 kg was purchased from Tee Group Films, Ladd, IL, USA.

### 2.2. Governing Equations

The description of various transport phenomena involves the solution of mass and energy conservation equations. The model equations contain four dependent variables and four governing equations. The four dependent variables were the fiber volume fraction ($V_{f}$), the plastic volume fraction ($V_{m}$), the void volume fraction ($V_{v}$), and mat thermal conductivity (k), W/(m.K). The four variables were a function of two independent variables, temperature T (K) and time t (s). The one-dimension energy balance equation is as follows [7]:

(1) $$\frac{\partial }{\partial t}\left[V_{f}C_{f}\rho_{f}+V_{m}C_{m}\rho_{m}+V_{v}C_{v}\rho_{v}\right]T=\frac{\partial T}{\partial z}\left(k\frac{\partial T}{\partial z}\right)-G$$

where, $C_{f}$, $C_{m}$, and $C_{v}$ are the specific heat capacity of the sorghum fiber cell wall, HDPE, and air in voids (J/(kg·K)), respectively. $\rho_{f}$, $\rho_{m}$, and $\rho_{v}$ denote the density of the sorghum fiber cell wall, HDPE, and air (kg/m^3^), respectively. *G* is the heat absorption rate of HDPE during a phase change. The energy balance equation indicates that the sum of energy consumption consumed to increase the mat temperature and the heat absorption due to the phase change of HDPE films should be equal to the heat conducted by contacting the hot-press plates. The HDPE phase change can also be represented as an equivalent internal energy change, and Equation (1) can be modified as follows [14]:

(2) $$\frac{\partial }{\partial t}\left[V_{f}C_{f}\rho_{f}+V_{m}C_{m}\rho_{m}+V_{m}C_{m}^{*}\rho_{m}+V_{v}C_{v}\rho_{v}\right]T=\frac{\partial T}{\partial z}\left(k\frac{\partial T}{\partial z}\right)$$

where $C_{m}^{*}\;$ is the apparent heat capacity of high-density polyethylene owing to the heat of fusion and was calculated later. $V_{f}$, $V_{m}$, and $V_{v}$ can be obtained as follows:

(3) $$V_{f}=m_{f}/(\rho_{f}*L*W*H)$$

(4) $$V_{m}=m_{m}/\left(\rho_{m}*L*W*H\right)$$

(5) $$V_{v}=1-V_{f}-V_{m}$$

where $m_{f}$ is the weight of sorghum fibers (kg), $m_{m\;}$ denotes the weight of HDPE (kg), *L* indicates the length of the mat (m), *W* is the width of the mat (m), and *H* is the thickness of the mat (m).

### 2.3. Testing and Simulation of HDPE Heat Capacity

The effective heat capacity of HDPE ($C_{m}^{’}$) was the sum of $C_{m}$ and $C_{m}^{*}$, and was measured by differential scanning calorimetry (Mettler-Toledo DSC 822), according to ASTM E1269-11 [16]. An empty aluminum holder, standard sapphire pellet, and HDPE film (20 mg) were sequentially examined in the temperature range of −20~200 °C at a heating rate of 20 °C/min and N_2_ gas flow of 50 mL/min. The sample was kept at −20 °C for 15 min in a liquid nitrogen environment to obtain a uniform temperature of the sample before testing. Two DSC scans were carried out for each material to ensure reproducibility. The $C_{m}^{’}$ was calculated according to the following:

(6) $$C_{m}^{’}=C_{p}\left(st\right)*D_{s}*W_{st}/\left(D_{st}*W_{s}\right)$$

where $C_{p}\left(st\right)$ is the specific heat capacity of the sapphire standard, and its value can be obtained from ASTM E1269-11. $D_{s}$ is the vertical displacement (mW) between the empty holder and the HDPE sample heat flow curves at a given temperature, and $D_{st}$ is the vertical displacement (mW) between the empty holder and the sapphire heat flow curves at a given temperature. $W_{st}$ and $W_{s}$ (mg) are the masses of the sapphire and HDPE samples, respectively.

The HDPE samples start to absorb heat during the melting stage, and the DSC heat flow outside of the melting temperate range reflects the specific heat capacity of HDPE. Therefore, the $C_{m}^{’}$ values obtained using Equation (6) in the temperature ranges of 0–100 °C and 160–200 °C were fitted by linear regression to obtain the $C_{m}$ value. The difference between $C_{m}^{’}$ and $C_{m}$ is $C_{m}^{*}$, and $C_{m}^{*}$ were fitted by a Gauss and a Lorentz equation.

### 2.4. Material Properties

#### 2.4.1. Heat Capacity of Sorghum Fiber and Air

The heat capacity of air ($C_{v}$) was considered to be constant (1000 J/(kg·K)). The heat capacity of sorghum fiber ($C_{f}$) in the oven-dried state was previously tested, and its prediction equation is as follows [16]:

(7) $$C_{f}=5.74T-469.1$$

#### 2.4.2. Thermal Conductivity

Oriented sorghum reinforced HDPE composites consisted of sorghum fibers, HDPE, and air in the voids. Their thermal conductivity of OFPC was measured and simulated in Qi et al. [4]:

(8) $$k=0.2\times 10^{-3}T+0.21\times 10^{-3}\rho +0.19V_{m}-0.21V_{m}^{2}-0.10$$

where ρ is the mat density (kg/m^3^) and $V_{m}$ is the mass ratio of HDPE, which ranged from 0 to 1.

### 2.5. Numerical Solution

Eight unknown variables (T, k, $C_{f}$,$\;C_{m}$,$\;C_{m}^{*}$,$\;V_{f}$,$\;V_{m}$, and $V_{v}$) and eight equations (Equations (2)–(5), (7), (8), (10) and (12)) were defined, allowing these equations to be solved. The energy equation (Equation (2)) is a nonlinear partial differential equation, and the initial and the boundary conditions must be defined to obtain its numerical solution. For this one-dimensional heat transfer model, one initial and two boundary conditions are required. The initial temperature ($T_{i}$) was ambient temperature of 25 °C. The Dirichlet boundary condition was employed to solve Equation (2) because the mat surface temperature rapidly increased to the target temperature. The boundary condition is as follows:

(9) $$T\left(0,t\right)=T\left(H,t\right)=T_{\infty }$$

where $T_{\infty }$ was the hot-press platen temperature (K). The known parameters are listed in Table 1. Equation (2) is a second-order nonlinear partial differential equation with varied heat capacity and thermal conductivity and no analytical solution [17]. Equation (2) was discretized over time and thickness variables; MATLAB^®^ software was used to obtain its numerical solution using the central-difference approximation for the derivative. The detailed numerical solution method and MATLAB code could be found in our previous study [18].

### 2.6. Experimental Evaluation of Heat Transfer

**Figure 1:**
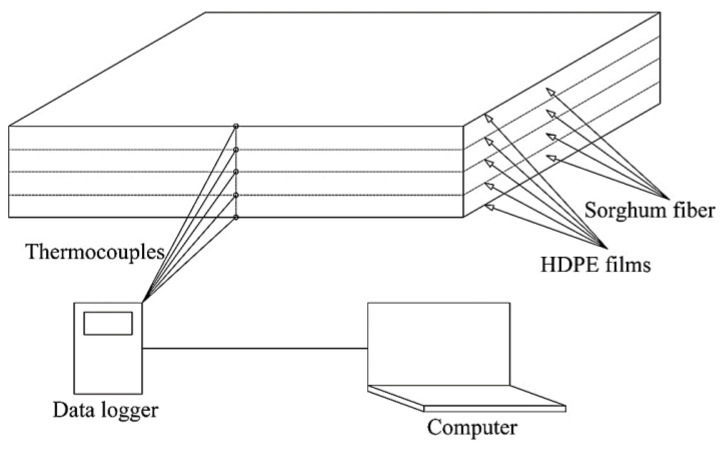
Schematic diagram of mat structure and thermocouple positions.

To better understand and simulate the effect of varying HDPE content, target mat density, and sorghum fiber moisture content on heat transfer through the mat during the hot-pressing process, an experimental design was devised, as shown in Table 2. The platen temperature was held constant in all cases at 160 °C. To study the influence of HDPE content in the mat on heat transfer, HDPE content values were changed while holding the target panel density and the moisture content of the panel constant at the values shown in Table 2. In order to precisely place the thermocouple (J type, EXTT-J-24-500, Omega, Norwalk, CT, USA) into the mat during hot-pressing, the OFPC fabrication method was modified from our previous study [1,2]. Sorghum fiber at a target moisture content level (as shown in Table 2) was evenly divided into four portions; each portion was formed by orienting the fibers with respect to the longitudinal axis, and pre-pressed at 35 MPa at room temperature for 4 min to obtain a layer of oriented sorghum fiber. HDPE films were divided into five portions (12.5%, 25%, 25%, 25%, and 12.5%). A portion of the HDPE film (12.5%) was first placed at the bottom, followed by placing a layer of sorghum fiber, and another portion of HDPE film (25%). The above steps were repeated till the second 12.5% HDPE film was placed on the top. All four layers of sorghum fibers were aligned in the longitudinal direction of the final mat, and each fiber layer was layered on both sides with 12.5% HDPE films. Double-sided silicone release papers were placed on the top and bottom of the mat in order to prevent HDPE from sticking to the metal caul plates during the hot-pressing process. Thermocouples were placed on the surfaces of the mat and in the middle of each layer of HDPE films (Figure 1). In this study, no adhesive or coupling agent was added in order to prevent heat release.

To fabricate OFPC, the mat of sorghum fibers and HDPE was hot-pressed between two platens heated to 160 °C for 10 min at a target thickness of 15 mm, followed by 30 min cold-pressing (cold water was piped into the hot-press plates). The mat temperature during hot-press was continuously recorded by a data acquisition system. Table 2 describes the experimental design to evaluate the influence of HDPE content, target density, and sorghum fiber moisture content on temperature distribution within the mat during the hot-pressing process. All the tests were repeated three times.

## 3. Results and Discussion

### 3.1. Heat Capacity and Heat Fusion of HDPE

**Figure 2:**
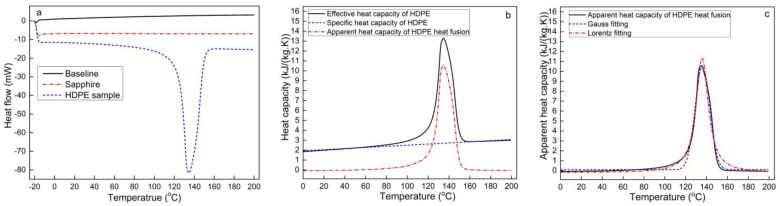
(**a**) Heat flow curves of heat capacity testing, (**b**) relationship between effective heat capacity and specific heat capacity of HDPE as well as the apparent heat capacity of the HDPE heat fusion, and (**c**) Gauss and Lorentz fits of the apparent heat capacity of the HDPE heat fusion.

The heat flow results for the empty aluminum holder, standard sapphire, and HDPE are shown in Figure 2a. The heat flows of the empty aluminum holder and standard sapphire changed linearly with temperature, while that of HDPE showed nonlinear changes. HDPE is a thermoplastic and exhibits endothermic behavior during melting. The onset temperature and ending temperature of HDPE melting were 121.2 °C and 151.3 °C, respectively, with a melting peak at 136.1 °C and a heat of fusion ($\Delta H_{f}$) of 180.2 J/g. The $\Delta H_{f}$ value obtained in this study is close to the $\Delta H_{f}$ of 178.6 J/g previously reported by Sotomayor et al. [19].

Figure 2b shows the $C_{m}^{’}$ calculated according to Equation (6). $C_{m}^{’}$ in the temperature ranges of 0–100 °C and 160–200 °C outside the HDPE melting temperate was considered as the specific heat capacity of HDPE and was fitted using the following linear equation:

(10) $$C_{m}=5.7\left(T-273.2\right)+1930.1$$

$$R^{2}=0.96$$

The $R^{2}$ value of Equation (10) was 0.96, and Figure 2b shows that Equation (10) predicts the baseline of $C_{m}^{’}$ well. The $C_{m}$ of HDPE in this study was found to be 2077.1 J/(kg·K) at 20 °C and 2899.1 J/(kg·K) at 170 °C based on Equation (10). These values are very close to the value of approximately 2000 J/(kg·K) at 20 °C previously reported by Oh [20]. Li et al. [21] reported that HDPE has a specific heat capacity of 2480 J/(kg·K) at 170 °C, which is lower than the value obtained in our study, possibly owing to the different molecular weight of the HDPE used in their study.

The apparent heat capacity of HDPE due to the heat of fusion was obtained by subtracting the $C_{m}$ from $C_{m}^{’}$, as shown in Figure 2b. Fits to Gauss and Lorentz functional forms were applied to model $C_{m}^{*}$, obtaining the following functions:

Gauss fitting:

(11) $$C_{m}^{*}=101.3+10486.4\exp\left(-0.01{\left(T-273.2-136.1\right)}^{2}\right)$$

$$R^{2}=0.98$$

Lorentz fitting:

(12) $$C_{m}^{*}=-197.8+2.3\times 10^{6}/\left[4{\left(T-273.2-135.8\right)}^{2}+198.8\right]$$

$$R^{2}=0.98$$

The $R^{2}$ values of the Gauss and Lorentz fits are both 0.98, showing that both equations fit the $C_{m}^{*}$ data very well. Figure 2c shows that the Gauss function describes the peak of $C_{m}^{*}$ better than the Lorentz function. The Lorentz function with no exponential component has a simpler form for numerical calculations, enabling the easy numerical solution of the energy equation. Thus, the Lorentzian function was used in later modeling.

### 3.2. Heat Transfer Simulation of Pure HDPE

**Figure 3:**
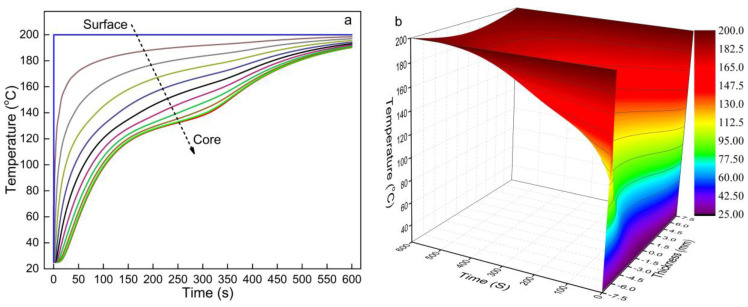
Heat transfer simulation results of pure HDPE at a hot-press temperature of 200 °C: (**a**) the change in temperature at different thicknesses and (**b**) 3D temperature distribution.

**Figure 4:**
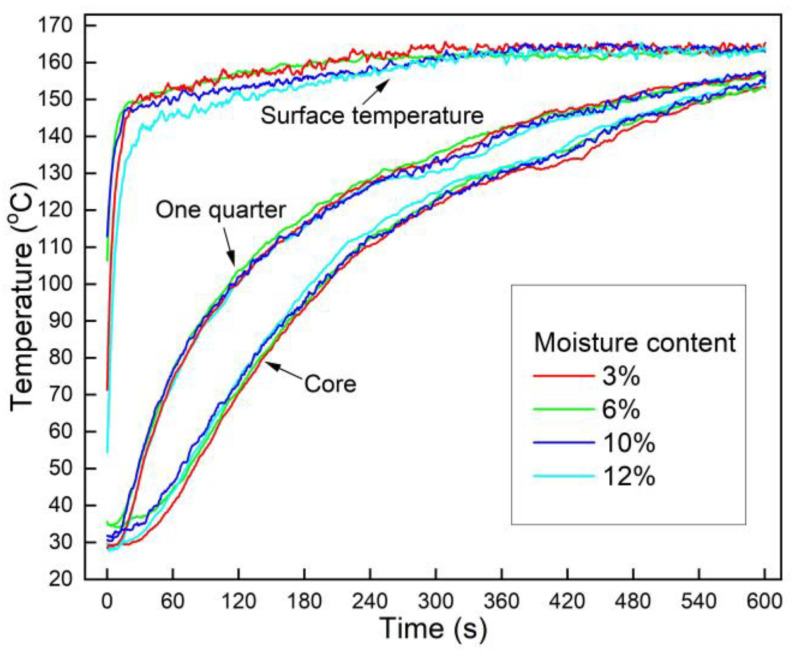
Effect of moisture content on heat transfer of OFPC during hot-pressing (the mat target density was 0.9 g/cm^3^ and the HDPE content was 10%).

To verify whether Equation (2) could be used to reflect the phase change characteristics of HDPE, $V_{m}$ was set to one when $V_{f}$ and $V_{v}$ were set to zero to simulate the heat transfer of pure HDPE. The initial temperature was 25 °C, $T_{\infty }$ was set as 200 °C, and the HDPE thickness was 15 mm for the simulation. Figure 4 shows the temperature changes over time at different thickness locations within the mat. Both Figure 3a,b show that the core temperature lags behind between 120 and 150 °C, corresponding to the melting of HDPE. Figure 3a,b also show that the temperature lag increased from the surface to the core of the HDPE mat, as more energy was absorbed to melt the HDPE closer to the core. The temperature increased with time because more energy was input through the heated hot-press platens. The simulated heat transfer of pure HDPE in this study is consistent with Woo et al. ’s research [14], and Equation (2) had a better prediction of the core temperature. Woo et al. [14] used the Gauss equation to describe the effective heat capacity of HDPE. Its prediction equation was divided into two segments with varied temperature factors to simulate HDPE heat transfer, which caused the predicted core temperature of HDPE during heating to abnormally increase above the melting peak temperature. However, the apparent heat capacity of HDPE predicted in Equation (11) is continuous and gradual, and it prevents an abrupt shift of the predicted temperature trend. These demonstrate that the $C_{m}^{*}$ can be used to replace the heat absorption rate of the HDPE phase change (*G*), and the $C_{m}^{*}$ of thermoplastics can be easily obtained from the DSC results. The use of $C_{m}^{*}$ also simplified the numerical solution of Equation (2), as a differential factor was omitted.

### 3.3. Effects of Moisture Content on Heat Transfer

Figure 4 illustrates the effect of moisture content on the core temperature and one-quarter position temperature, and no obvious impact was observed. Owing to the closure of the hot press platens, the mat surface temperature rapidly increased to 150 °C in 20 s and can be described using the Dirichlet boundary condition. The surface temperature continued to increase to 160 °C during the next 300 s as the platens gradually reached the set target temperature. Additionally, the HDPE films at the surface absorbed energy during the melting process, decreasing the surface temperature. At the one-quarter position below the mats’ surface, the temperature was much higher than that at the core, and this temperature difference decreased with time. The heat was transferred from the higher temperature region to the lower temperature one, and the heat gradually transferred from the surface to the core.

The temperature at the core and the one-quarter position did not obviously change when the moisture content of sorghum fiber increased from 3% to 12%, indicating that the fiber moisture content had little impact on the heat transfer of OFPC. Furthermore, unlike in a traditional natural fiber-based mat with liquid thermosetting resin, the HDPE films in the mat acted like barriers for water vapor flowing through the mat thickness. Therefore, it was reasonable to exclude the heat convection of vapor in the heat transfer model of the OFPC.

### 3.4. Effects of Mat Density on Heat Transfer

**Figure 5:**
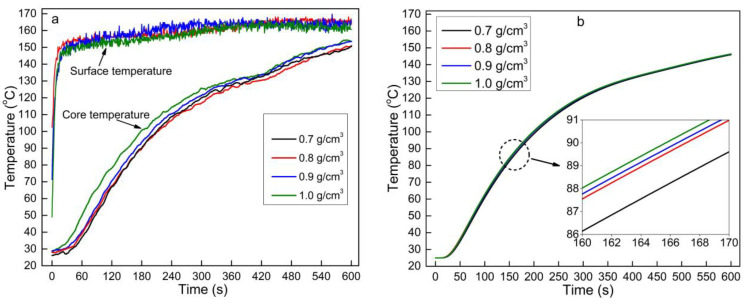
Effects of mat density on the mat core temperature during hot-pressing: (**a**) measured data and (**b**) modeling data (the sorghum fiber moisture content was 3% and HDPE content was 10%).

Figure 5 shows the effects of mat density on the mat core temperature during OFPC hot-pressing. The mat core temperature was higher at a higher mat density in both the experimental test (Figure 5a) and the mathematic model (Figure 5b). A close examination of Equation (8) shows that the thermal conductivity of the mat linearly increases with density, and a higher thermal conductivity results in higher efficiency of thermal conduction, supporting the experimental results that indicate an increase in the core temperature with increasing mat density. The mat contained more sorghum fiber and HDPE content per unit volume at a higher density. These materials, including more molecules in the mat, absorbed more energy to increase their internal energy. This had an inverse effect on the temperature increase of the mat, and Equation (2) also supports the inverse impact of mat density. The temperature increase due to energy absorption depends on the specific heat capacity. The mat core temperature increased with density under the combined effects of a higher heat transfer efficiency and higher heat absorption at a higher mat density.

### 3.5. Effect of HDPE Content on Heat Transfer

**Figure 6:**
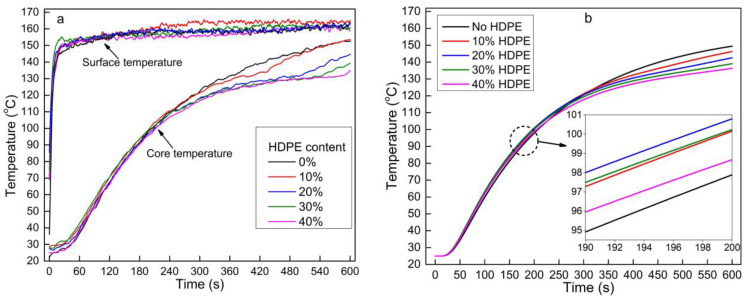
Effects of HDPE content on the mat core temperature during hot-pressing: (**a**) measured data and (**b**) modeling data (the sorghum fiber moisture content was 3% and mat target density was 0.9 g/cm^3^).

Figure 6 illustrates the effect of HDPE content on the core temperature of the OFPC mat during hot-pressing. No temperature lag was observed in the experiment (Figure 6a) when HDPE was not added to the mat. However, the core temperature lagged between 300 and 480 s when 10% HDPE was incorporated into the mat. A higher HDPE content resulted in a lower core temperature after reaching the melting temperature of HDPE. HDPE is a thermoplastic and phase-change material that absorbs heat during melting; thus, more heat was absorbed with a higher HDPE content, which delayed the increase in the mat core temperature during melting. A similar trend was found in the mathematic modeling results (Figure 6b). The mat core temperature rapidly increased after all the HDPE melted (Figure 6a), as no additional heat was required to melt HDPE.

During the initial stage of hot-pressing (below 100 °C), the core temperature of the mat with HDPE was higher than the mat without HDPE (Figure 6b), as the HDPE had a higher thermal conductivity (0.44 W/(m.K) at room temperature) and a higher thermal conduction efficiency than sorghum fiber (0.12–0.2 W/(m.K)). According to Equation (8), the thermal conductivity of the OFPC mat non-linearly increases with the HDPE content, and a higher thermal conductivity helps the thermal conduction from the surface to the core. In addition, the specific heat capacity of HDPE (2044.1 W/(m.K) at 20 °C) was much higher than that of the sorghum fiber (1213.9 W/(m.K) at 20 °C) [18]. Therefore, the mat with a higher HDPE content required more time to reach the same temperature at the same energy input. Owing to the combined effects of higher thermal conductivity and specific heat capacity of the mat at a higher HDPE content, the core temperature of the OFPC mat first increased with the HDPE content (0 to 20% HDPE), and then decreased at higher HDPE contents (30% and 40% HDPE) (Figure 6b). This trend was not obvious in the experimental results (Figure 6a), possibly owing to temperature fluctuations of the hot-press platens and an experimental error.

### 3.6. Temperature Distribution Prediction

**Figure 7:**
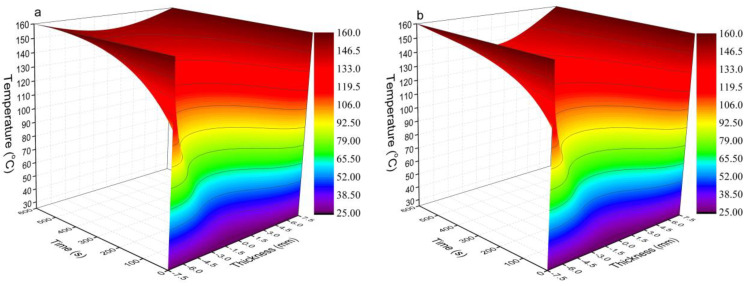
Heat transfer prediction results during the hot-pressing of sweet sorghum composites (**a**) without HDPE and (**b**) with 40% HDPE (the mat target density was 0.9 g/cm^3^).

Figure 7 shows the temperature prediction results of the OFPC mat during hot-pressing without HDPE (Figure 7a) and with 40% HDPE (Figure 7b). The temperature increased from the core to the surface and increased with time whether or not the mat contained HDPE. The surface temperature remained at 160 °C as the Dirichlet boundary condition was applied. After 10 min of hot-pressing, the core temperature of the mat without HDPE was 149.4 °C (Figure 7a), and it was 136.3 °C for that with 40% HDPE (Figure 7b). This 13.1 °C difference indicates that it is necessary to increase the temperature during the consolidation process and/or extend the hot-pressing duration to further increase the core temperature in the OFPC containing 40% HDPE, as a core temperature of 136.3 °C is not sufficient for HDPE to flow easily.

### 3.7. Comparison of Experimental Results with the Model Prediction

**Figure 8:**
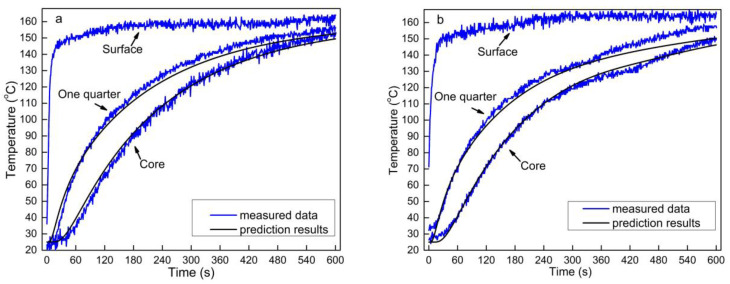

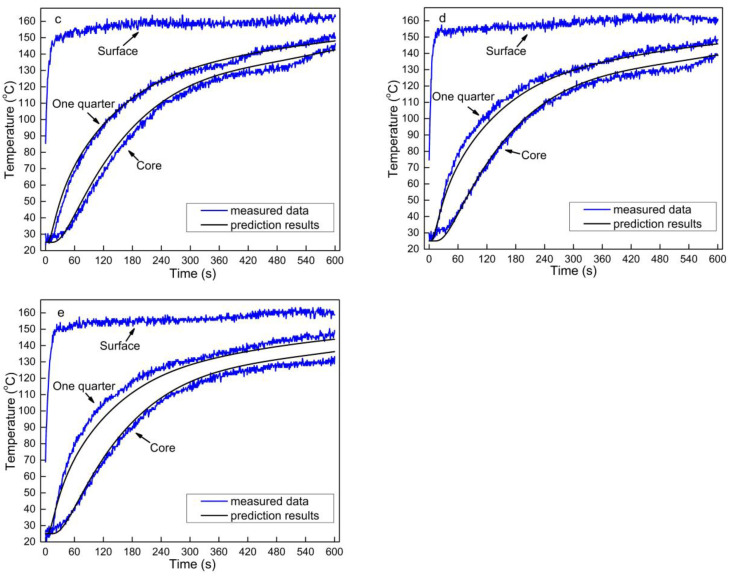
Comparison of heat transfer measured data of sweet sorghum fiber composites with predicted results: (**a**) without HDPE content, (**b**) 10%, (**c**) 20%, (**d**) 30%, and (**e**) 40% HDPE content (the sorghum fiber moisture content was 3% and mat target density was 0.9 g/cm^3^).

Figure 8 compares the measured temperatures at various locations within the OFPC mats with varying HDPE content during hot-pressing with those predicted by the model. In general, the predicted temperature at both the core and one-quarter positions was similar to that of the experimental values. The predicted temperature at the one-quarter thickness was slightly lower than that of the measured temperatures beyond 100 °C when no HDPE was added to the mat (Figure 8a). This could be because of convection heat transfer as there was no HDPE layer to act as a barrier preventing the transfer of water vapor from the surface to the core. However, in modeling this behavior, the energy equation (Equation (2)) did not consider the heat transfer due to convection resulting from the 3% moisture content of sorghum fiber.

A temperature lag was observed in the measured data between 120 and 140 °C when HDPE was incorporated into the OFPC (Figure 8b–e), but this phenomenon was not obvious in the model perdition, mainly because the HDPE in the OFPC was not evenly distributed and was present in layers. Additionally, controlling the temperature of the hot platens, thermocouple position, experimental test error, and model hypothesis also affected the difference between the measured data and model prediction data.

### 3.8. Optimization of Hot-Press Parameters

**Figure 9:**
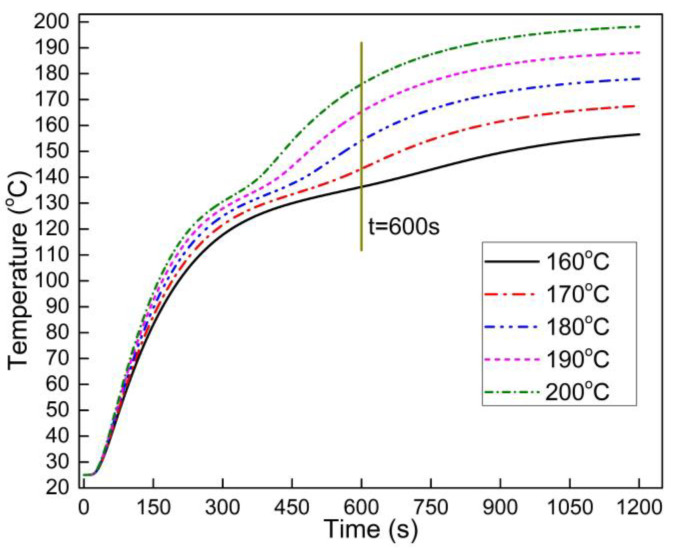
Core temperature simulation results of OFPC with 40% HDPE under different hot-press temperatures (the mat target density was 0.9 g/cm^3^).

The most important function of mathematic modeling is to guide the manufacture of composite panels. As the onset and ending melting temperature of HDPE are 121.2 °C and 151.3 °C, respectively, the core temperature of the OFPC must reach at least 151.3 °C. The above analysis showed that a hot-press temperature of 160 °C and a duration of 10 min were not sufficient to attain the required core temperature to hot press a panel with 40% HDPE as the maximum core temperature was 136.3 °C. Figure 9 shows the predicted core temperature of OFPC containing 40% HDPE hot-pressed at 160 °C, 170 °C, 180 °C, 190 °C, and 200 °C for 20 min. A higher hot-pressing temperature and longer duration resulted in a higher core temperature. As a higher hot-pressing temperature consumes more energy and a lower hot-press duration results in higher productivity, the hot-pressing parameters should balance these two factors. Table 3 provides the predicted hot-pressing durations of OFPC at various HDPE contents when the core temperature reached 151.3 °C. A press temperature of 180 °C and a duration of 443 s were recommended for OFPC with 10% HDPE, and 180 °C and 574 s for OFPC with 40% HDPE.

## 4. Conclusions

A one-dimensional heat transfer model of natural fiber reinforced thermoplastic composites during hot-pressing was established. The novelty of this study is that the apparent heat capacity of thermoplastics was first simulated and then coupled with the heat transfer model to simulate the temperature distribution of natural fiber reinforced thermoplastics composites during hot-pressing. The heat transfer results predicted by the mathematical model were consistent with the experimental data. The moisture content of sorghum fiber had little effect on the heat transfer of OFPC. Both the experimental and model results showed that the mat core temperature slightly increased with the mat density. The addition of HDPE retarded the temperature increase after reaching its melting temperature, and a greater HDPE content resulted in a lower core temperature. A higher press temperature or a longer press time was required to fabricate an OFPC with a higher HDPE content. The thermal conductivity efficiency was higher at higher HDPE contents. A predictive tool was developed to assist manufacturers of OFPC panels in setting the processing parameters, namely press temperature and duration, depending on the HDPE content. The limitation of the heat transfer model in this study is that it does not consider the heat flow of molten thermoplastics.

## Figures and Tables

**Table 1:** Parameters and values of the mathematical model.

Parameters	Symbols	Values	Unit
Specific heat capacity of air	$C_{v}$	1000	J/(kg·K)
Density of sorghum fiber cell wall	$\rho_{f}$	1500	kg/m^3^
Density of high-density polyethylene	$\rho_{m}$	940	kg/m^3^
Air density	$\rho_{v}$	1.225	kg/m^3^
Thermal conductivity of HDPE	$k_{m}$	0.44	W/(m·K)
Mat thickness	H	0.015	m

**Table 2:** Experimental design for the effects of HDPE content, mat density, and sorghum fiber moisture content on heat transfer of the mat during hot-pressing.

Variables	Values	Fixed Parameters	Platen Temperature
HDPE content	0, 10, 20, 30, 40%	Target mat density was 0.9 g/cm^3^, 3% moisture content of sorghum fiber	160 °C
Mat density	0.7, 0.8, 0.9, 1.0 g/cm^3^	10% HDPE, 3% moisture content of sorghum fiber	
Sorghum fiber moisture content	3, 6, 9, 12%	10% HDPE, target mat density was 0.9 g/cm^3^	

**Table 3:** The predicted hot-press duration of OFPC with varied HDPE content when the core temperature reached 151.3 °C.

Hot-Press Temperature (°C)	Hot-Pressing Duration (s)				
	No HDPE	10% HDPE	20% HDPE	30% HDPE	40% HDPE
160	645	710	781	861	954
170	490	533	582	637	702
180	410	443	480	523	574
190	360	385	415	451	494
200	323	344	370	400	437

## Data Availability

The data presented in this research are available on request from the corresponding author.
